# Supplementary material for: Patterns of Intron Gain and Loss in Fungi
Source: PLoS Biol. 2004 Nov 30;2(12):e422. doi: 10.1371/journal.pbio.0020422 (PMC532390; doi:10.1371/journal.pbio.0020422)
Supplement: Table S1 — Also available at http://genes.mit.edu/NielsenEtAl/. (4.3 MB ZIP). [file pbio.0020422.st001.zip › NielsenEtAl/html/536.html]

AN2368.1.NCU05735.1.MG05435.1.FG04426.1


```
 CLUSTAL W (1.82) Multiple Sequence Alignments - Introns Inserted


Sequence 1: AN2368.1	520 aa
Sequence 2: NCU05735.1	554 aa
Sequence 3: FG04426.1	558 aa
Sequence 4: MG05435.1	564 aa
Alignment Length: 582 aa
Number Identitical Residues: 167 aa
Alignment Score (without introns) 10253


MG05435.1 	------------------MSRSSPQPGAA~SPTDVPTAPITADDSVPAPAPAGITEEELE
NCU05735.1	-----MGQQTLEQQQQQQPHHQQPQ-QHP~ESSECES-PSQAQQDVQVDATT---EFDLD
FG04426.1 	MSSPQASLPFSTQQRFRVSFCQRVLRRIR2MAVDVMSRPDEENTGVRAGDGSRDSSVDVE
AN2368.1  	-----MAIQFDVNKRRETSLEAGVQNEKP~DPNASAV-GTESPPPFLLDKE------SLD
          	      .     ..                 .             .           .::

MG05435.1 	RLGRQRPAAFSSWWHEWCFCGSILGSMLMA~EFFISGFIIVLPNVSQSINMPPGTQTWPA
NCU05735.1	RVGRQRPAVFSSTLEEVMFCGSILISMLMS0EYFVSGFNIILPGLAGALDIPKNSQTWPS
FG04426.1 	KLGRQRPEVFKSIWSELGFGFSVFCSMLLA0EFFVSGFHVILPPLSEELEIPAASQTWPS
AN2368.1  	RLGRERPDVFPNRWIELGFCFCLLSSELLA0EYFVSGFNNLLPHITAALDIPPGSQTWPA
          	::**:** .* .   *  *  .:: * *:: *:*:***  :** ::  :::*  :****:

MG05435.1 	SVFTLVTGALLFAFGRIADMYGGRTVFLFGMLWYTLWIFIAGFCSNHVALIVARAMQGIG
NCU05735.1	SVFSLVTGSFLLPFGRLADIYGAYFIFTGGLVWMCIWCLIGGFSQNYQMLIVTRALAGLG
FG04426.1 	SVFSLVTGAMLLPMGRLGDMYGGYLVYNAGLLWFFLWALIAGFSKNYMMLIFCRALQGLG
AN2368.1  	SVFSLVTGACLLPIARLADIYGPRLVFNAGLIWYIVWSFIAGWSVNYKMLVVCRALQGLG
          	***:****: *:.:.*:.*:**   ::  *::*  :* :*.*:. *:  *:. **: *:*

MG05435.1 	PAAFLPSGVMLLGSMYRPGPRKNMVFSIYGSFAPIG~FFVGTLAGG~ALAEFLDWRWYFW
NCU05735.1	PAAFLPTGLMLLGKTYRPGPRKNLVFSLYSAFAPLG~FFIGIILGG~VTGEYLSWRWWFF
FG04426.1 	PAAYLPGGVMLMGKIYRPGPRKNLVFALYGSFAPLG1FFLGIFVGG~LSAEFLNWRWYFW
AN2368.1  	PAAFLPAGIMLIGSIYRPGPRKNLVFSLYGAFSPVG~FYGGICVSG2LS-----------
          	***:** *:**:*. ********:**::*.:*:*:* *: *   .*              

MG05435.1 	IGTIILFLISVASFFAIPKEFKDRPVSKEGKAE~MKMDWLGVATILPGLLLFVFAITQGA
NCU05735.1	IGTIILFVTSLMAYLTIPNDRHQAVA---TRGG~AKMDYFGLFTIVPAIILITFAITTGG
FG04426.1 	IGTIIFAVVCGISLVAVPFDYRD------RKVD~IPMDWWGVGTIVPGLLLIVYSLTDSS
AN2368.1  	-------------------DSGRRPG---RLPD1LKMDWWGCCTIVPGLILLVFAVTDGS
          	                   :                **: *  **:*.::*:.:::* ..

MG05435.1 	HAPQGFATPYVYVTLILGVLILGCAVYVEGWVAEQPLV2PFDLFAPKYMTPMVLGLFNIY
NCU05735.1	SAPNGWRTPYIPVTFVIGMLFLCAAIYLEGWVAEQPLL~PKDLFAAKYMKRFTLALFFAY
FG04426.1 	HAPNGWATPYIIVTLILGVMFLTAAWYVETHKAQVPLL~PADLFEPKGMKRFLAVLFMAF
AN2368.1  	HAPDGWRTPYVLVTFLLGIALLAAAFYIEGWVASEPLL~PFDVFKVKSMSPLFVALSFSY
          	 **:*: ***: **:::*: :* .* *:*   *. **: * *:*  * *. :   *   :

MG05435.1 	GSFGIFQFYASFY2IQEVIGHPPLLTAAWFTPMAVGGTILAVMGGIVLHRIPSHLLLVIS
NCU05735.1	GVFGIFLFYASVH2IEAFMGASTLQTATWFAPMAGGGIVLATIGGFTLHKLPGRVLLMIS
FG04426.1 	GTFGLFLFYSSFY~MELVLHRSPLTTAVWYIPMCVGGLIIGTVGGFTLHFLPGRVLLFIS
AN2368.1  	GVFGIYLFYASFY2IQDILGHNSLISAAWFAPMAAGGVILATVGGFTLHHLPGKLLLLIS
          	* **:: **:*.: :: .:   .* :*.*: **. ** ::..:**:.** :*.::**.**

MG05435.1 	AAGSLASVLLFALIPSDPAALNYWAWVFTAMIGGTLGIDISFNISSVFMTTALPSHRQGL
NCU05735.1	GGGCLASVLLFALIPDHA---NYWAFVFPAMLGATIGVDITYLVSNVFITTNVPAHMQGT
FG04426.1 	SMANAICMLLFALMPENP---NYWAWVFPSMVCCTLGIDITFTVSNIFLTTNMPSHRQGL
AN2368.1  	GAGYLVSMLLFAIIPDNP---NYWAYVFPAMVAATVGVDIGYSVSNIFITTNLPQNRQGV
          	. .   .:****::*...   ****:**.:*:  *:*:** : :*.:*:** :* : ** 

MG05435.1 	AGALINTLVFLGIGFYLGLADLIVESTKVGDADKDKAEGFKVAFWLGVGCSATALVIATL
NCU05735.1	AGALINSLVFIGISFFLGIADLAVAES----EDAFDGENHRVAFWFAVACAACSLLLFCL
FG04426.1 	AGALINSVLFLGISFFLGIADIAVAET----SELGLRKSYKVAFWLAFGVASIPLLFIPF
AN2368.1  	AGAIINTIVFAGISFFLGLADLVVAET----KDLGAAGSYKAAFWFGVACSGVAILLLVF
          	***:**:::* **.*:**:**: * .:     :     ..:.***:... :. .:::  :

MG05435.1 	FTRIGRMKSDLTFDEKMELEAEAARLQTCGAGGQSETSSGPKKNDGSGS
NCU05735.1	ID-PGKAESELTLEERMEAG-LGPKKETSASGSESPNPEQESE------
FG04426.1 	IN-IGSAKSDLTMEERQR----LENPQRSTEEPERDREAKTSA------
AN2368.1  	IR-VGKAQSDLTVEERIQ---LAASLAVGDVTPEQLAAAGHSS------
          	:   *  :*:**.:*: .               :       .
```
